# Management of patients with acute ST-segment elevation myocardial infarction in Russian hospitals adheres to international guidelines

**DOI:** 10.1136/openhrt-2019-001134

**Published:** 2020-01-23

**Authors:** Anna V Kontsevaya, Katie Bates, Henrik Schirmer, Natalia Bobrova, David Leon, Martin McKee

**Affiliations:** 1 Department of Non-Communicable Disease Epidemiology, National Research Centre for Preventive Medicine, Moscow, Russian Federation; 2 Department of Medical Statistics, Informatics and Health Economics, Medical University Innsbruck, Innsbruck, Austria; 3 Department of Health Services Research and Policy, London School of Hygiene and Tropical Medicine, London, United Kingdom; 4 Division of Medicine and Laboratory Sciences, Institute of Clinical Medicine, The Arctic University of Norway UiT, Tromsø, Troms, Norway; 5 Department of Non-communicable Disease Epidemiology, London School of Hygiene and Tropical Medicine, London, UK

**Keywords:** primary PCI, STEMI, public health

## Abstract

**Objective:**

Russia has one of the highest cardiovascular mortality rates. Modernisation of the Russian health system has been accompanied by a substantial increase in uptake of percutaneous coronary intervention (PCI), which substantially reduces the risk of mortality in patients with acute ST-elevation myocardial infarction (STEMI). This paper aims to describe contemporary Hospital treatment of acute STEMI among patients in a range of hospitals in the Russian Federation.

**Methods:**

This study used data from a prospective observational cohort of 1128 suspected patients with myocardial infarction recruited in both PCI and non-PCI hospitals across 13 regions and multiple levels of the health system in Russia. The primary objective was to examine the use of reperfusion strategies in patients with STEMI.

**Results:**

Among patients reaching PCI centres within 12 hours of symptom onset, the vast majority received angiography and PCI, regardless of age, sex and comorbidity, in line with current European Society of Cardiology guidelines.

**Conclusion:**

Patients reaching Russian hospitals are very likely to receive appropriate treatment, although performance varies. The best hospitals can serve as beacons of good practice as PCI facilities continue to expand across Russia where geography allows.

**Key questions**

**What is already known on this subject?**

- The management of ST-elevation myocardial infarction (STEMI) has been transformed by the introduction of modern methods for reperfusion, notably percutaneous coronary intervention (PCI), use of which has been increasing in Russia.
- Little is known about individual-level patient characteristics determining associated with use of PCI in Russia, or how PCI use varies across different levels of the health system with PCI facilities in Russia

**What does this study add?**

- In PCI capable centres, we demonstrate that the vast majority of patients are treated in line with European Society of Cardiology guidelines, regardless of patient characteristics. However, angiography and PCI use do vary across different levels of the health system.

**How might this impact clinical practice?**

- There is an opportunity to reduce the variation in treatment of patients with STEMI in PCI-capable hospitals across levels of the Russian health system. There are hospitals in Russia that can serve as beacons of good practice to facilitate this standardisation of care for patients with STEMI.

## Introduction

This paper describes the management of patients with ST-elevation myocardial infarction (STEMI) in a selection of hospitals across the Russian Federation, a country that has received relatively little attention in the international cardiological literature, but which has a burden of cardiovascular disease (CVD) that is among the highest in the world^1^ and which has seen major investments in treatment of CVD in recent years.

The management of STEMI has been transformed by the introduction of modern methods for reperfusion. Treatment must, however, be given rapidly, before hypoxia causes permanent damage to the myocardium. The European Society of Cardiology (ESC) guidelines advocate percutaneous coronary intervention (PCI) by an experienced team for all patients within 12 hours of symptom onset (early phase of STEMI).^2–4^ This depends on there being a well-functioning, adequately resourced health system.^5^ Modernisation of the Russian health system has been accompanied by a substantial increase in uptake of PCI in all parts of the country, although to a greater extent in some regions than in others.^6^ This investment is thought to have contributed, at least in part, to the sustained decline in mortality since 2005. However, treatment intensity remains below that in many other countries^7^ and there are considerable challenges in providing rapid access to advanced care in such a vast and sparsely populated country.^8^ An effective response to these challenges will require an understanding of how the system is working.

There has been only limited research on management of acute myocardial infarction (AMI) in Russia so far^9–11^ and relatively little is known about what happens across different types of medical facilities accepting AMI patients in Russia. This paper reports results from the first study in Russia designed specifically to assess contemporary treatment of AMI across multiple levels of the health system and whether treatment varies by patient characteristics.

## Data and methods

The protocol for the 'Management of Acute Myocardial Infarction in Russia’ (MAMIR) Study, part of the International Project on Cardiovascular Disease in Russia (IPCDR), has been published previously.^12^ In brief, MAMIR is a prospective observational study describing the current treatment of AMI in Russia. A random sample of 1128 patients, aged 35–75 years, with a confirmed AMI diagnosis, and surviving 24 hours after admission to the hospital, within 16 hospitals across 13 regions of Russia from 2015 to 2017, were recruited. Hospitals were selected using purposive sampling, to include all types of hospital, large and small, and be willing to commit to organising data collection over the entire study period and covering regions of different levels of economic development from a large part of the territory of Russia. Of the 16 hospitals included, all were able to perform medical reperfusion therapy and 12 were able to provide PCI. For all hospitals, 2015 data including number of patients with MI, mortality rates and PCI rates were collated from official statistics.^13^

Data for individual-level analyses came from patient interviews and abstracted medical records regarding the patients’ initial index admission. Core data include age, sex, STEMI status (from ECG), reperfusion treatments, admission characteristics including date and time of admission, pre-hospital delay to hospital (<12 hours, 12–48 hours, >48 hours), use of emergency medical services (EMS) and whether patients came directly to the hospital or via another health facility.

To capture actual practice, patients with STEMI were identified using an intention-to-treat approach, defined as having a STEMI if ST-elevation was found on their first ECG taken after the onset of MI symptoms (n=777). Appropriate treatment was determined with reference to the 2014 ESC guidelines, which were the guidelines adopted in Russia when data collection began. We have updated our nomenclature to match the 2017 guideline terms, as the 2017 revisions have no material implications for classifying treatment strategies for patients in our study. Specifically, we have categorised patients with STEMI by time from symptom onset to first medical contact (early phase, evolved and recent STEMI), the treatment indicated for each stage is described in online supplementary web appendix, figure 1.

10.1136/openhrt-2019-001134.supp2 Supplementary data

PCI hospitals were divided into Federal, Regional or City level, corresponding broadly to decreasing levels of complexity of services. In PCI hospitals, age-standardised rates of (1) diagnostic coronary angiography (CA), first, initial reperfusion attempt of either (2) thrombolytic therapy (initial TLT) or (3) PCI (initial PCI) and (4) ultimate PCI use (any PCI use including initial PCI and rescue PCI following failed TLT) were reported, using the 2013 European Standard population. Differences in use by age, sex, pre-hospital delay and hospital level were tested with Pearson χ^2^ test and Fisher’s exact test where appropriate.

Further analyses were stratified by STEMI stage and type of hospital (PCI or non-PCI capable) to assess differences in AMI management by patient (age, sex and comorbidity) and admission characteristics (direct/indirect routes to hospital of definitive treatment; use of EMS). Associations between patient and admission characteristics with angiography, TLT and PCI use among patients with early phase STEMI (n=452), and angiography and PCI use in patients with evolved (n=125) and recent (n=55) STEMI in PCI hospitals, were modelled using logistic regression.

Basic descriptive analyses of patients admitted to a non-PCI hospital, and remained 24 hours after admission, were undertaken by STEMI phase.

## Results

### Hospital characteristics

Summary characteristics of the hospitals included in the MAMIR Study are available in table 1. Additional details are available in the previously published protocol paper.^12 13^

**Table 1:** Characteristics of patients by PCI/non-PCI hospital in MAMIR Study

		PCI centre	Non-PCI centre	Total	χ^2^, p-value
Age (years)					
35–59	n	483	65	548	0.03, 0.872
	%	48.5	49.2	48.6	
60–75	n	513	67	580	
	%	51.5	54.5	51.4	
Sex					
Female	n	224	32	256	0.20, 0.651
	%	22.5	24.2	22.7	
Male	n	772	100	872	
	%	77.5	81.3	77.3	
STEMI					
No	n	267	78	345	56.43, <0.001*
	%	26.8	59.1	30.6	
Yes	n	723	54	777	
	%	72.6	40.9	68.9	
Missing	n	6	0	6	
	%	0.6	0	0.53	
Transported directly†					
No	n	312	66	378	18.24, <0.001
	%	31.3	50	33.5	
Yes	n	684	66	750	
	%	68.7	50	66.5	
Transported by EMS					
No	n	301	69	370	25.71, <0.001
	%	30.2	52.3	32.8	
Yes	n	695	63	758	
	%	69.8	47.7	67.2	
Pre-hospital delay‡					
<12 hours	n	590	52	642	68.51, <0.001
	%	59.2	39.4	56.9	
12–48 hours	n	175	25	200	
	%	17.6	18.9	17.7	
>48 hours	n	79	41	120	
	%	7.9	31.1	10.6	
Missing	n	152	14	166	
	%	15.3	10.6	14.7	
Total	n	996	132	1128	
	%	88.3	11.7	100	

*Fisher’s exact test

†Directly to hospital of definitive treatment (where patient in analysis sample was both finally treated for suspected AMI and recruited to study).

‡Delay from symptom onset to admission to hospital of definitive treatment.

AMI, acute myocardial infarction; EMS, emergency medical service; MAMIR, Management of Acute Myocardial Infarction in Russia; PCI, percutaneous coronary intervention; STEMI, ST-elevation myocardial infarction.

Across PCI hospitals, use of PCI within 24 hours of admission varied from a low of 20.5% in Rostov to 87.8% of patients in Belgorod, the hospital which also had the lowest inpatient mortality among patients with AMI. Bryansk had the highest inpatient mortality; it did not provide PCI in 2015 but has since become a PCI hospital.

### Patient characteristics

In all, 1128 patients were recruited to the MAMIR Study, the majority from PCI hospitals. 77.3% were men with mean age 57.7 years; mean age of women was 62.3. 68.9% of patients had a STEMI; nearly three-quarters of patients recruited at PCI hospitals had STEMI compared with 40% of patients with AMI at non-PCI hospitals (table 1).

Almost 60% of all patients arrived within 12 hours of symptom onset, but only 40% in non-PCI hospitals; almost a third of patients in these hospitals arrived after 48 hours. Two in three patients travelled directly to their hospital of definitive treatment, the proportion was greatest among those patients recruited in PCI hospitals (table 1). Two-thirds of patients came via EMS; a higher proportion of patients in PCI hospitals were transported by EMS.

### STEMI patients

Of the 777 patients with STEMI, the vast majority were recruited at PCI hospitals. Most patients were men and just over half were under 60-year old (table 2). Data on pre-hospital delay were available for 88% of patients; these complete cases had near identical proportions of patients by age and sex as the full sample of patients with STEMI. Nearly three-quarters of patients admitted to PCI hospitals were early phase STEMI, thus eligible for PCI (in the absence of contraindications); in non-PCI hospitals just under half of patients arrived within 12 hours (table 2).

**Table 2:** Patients with STEMI by age, sex and pre-hospital delay by PCI/non-PCI hospital in MAMIR Study

		PCI hospitals					Non-PCI hospitals				
		<12 hours	12–48 hours	>48 hours	Total	χ^2^, p-value	<6 hours	<6–12 hours	12+hours	Total	χ^2^, p-value
Sex											
Female	n	99	22	11	132	1.13, 0.570	3	1	4	8	0.46, 0.881*
	%	75.0	16.7	8.33	100		37.5	12.5	50.0	100	
Male	n	353	103	44	500		11	8	22	41	
	%	70.6	20.6	8.80	100		26.8	19.51	53.7	100	
Age (years)											
35–59	n	229	72	29	330	1.89, 0.388	6	7	16	29	2.83, 0.264*
	%	69.4	21.8	8.79	100		20.7	24.14	55.2	100	
60–75	n	223	53	266	302		8	2	10	20	
	%	73.8	17.6	8.61	100		40.0	10.0	50.0	100	
Total	n	452	125	55	632	–	14	9	26	49	–
	%	71.5	19.8	8.7	100		28.6	18.4	53.1	100	

*Fisher’s exact test.

MAMIR, Management of Acute Myocardial Infarction in Russia; PCI, percutaneous coronary intervention; STEMI, st-elevation myocardial infarction.

Age-standardised rates reveal that a very high proportion of patients with STEMI in PCI hospitals received angiography; the proportion for men was slightly higher than women (tables 3 and 4). Among patients receiving CA who received subsequent PCI, this sex gap widened, nearly all men received PCI but only three-quarters of women. This gap was seen at all levels of the health system. Compared with Federal and Regional hospitals, PCI use following CA was lower among both men and women in City hospitals; the sex gap remained, nearly twice as many men received PCI following CA as women (table 3).

In the subsequent sections, we examine the extent to which these differences can be explained by other factors.

**Table 3:** Age standardised angiogram, PCI and TLT rates for patients with STEMI in All PCI hospitals, and by type of PCI hospital by sex

	ALL hospitals (*95% CI*)		ALL PCI hospitals (*95% CI*)		PCI hospitals (*95% CI*)					
					Federal		Regional		City	
	M	F	M	F	M	F	M	F	M	F
Angiogram	86.4 (81.9 to 91.0)	83.0 (72.4 to 93.7)	91.40 (87.3 to 95.5)	87.31 (77.0 to 97.6)	98.03 (96.4 to 99.7)	80.75 (73.9 to 87.7)	95.49 (91.6 to 99.4)	82.20 (77.9 to 86.5)	58.05 (47.2 to 68.9)	39.42 (33.0 to 45.8)
PCI (first)	–	–	57.8 (51.5 to 64.2)	54.9 (47.3 to 62.6)	72.9 (67.1 to 78.7)	37.4 (28.9 to 45.9)	52.1 (42.8 to 61.4)	71.3 (64.5 to 78.2)	42.0 (30.5 to 53.5)	17.2 (9.9 to 24.5)
PCI (first) (% of those having angiogram)	–	–	63.3 (56.6 to 69.9)	59.5 (52.0 to 67.0)	74.1 (68.2 to 79.9)	42.7 (37.5 to 47.8)	53.9 (44.3 to 63.4)	74.7 (68.0 to 81.3)	63.2 (52.8 to 73.7)	21.9 (11.7 to 32.0)
TLT (first), All patients*	33.1 (272 to 39.1)	27.3 (15.1 to 39.5)	33.8 (27.7 to 39.9)	28.4 (16.0 to 40.8)	22.5 (16.9 to 28.0)	33.0 (22.3 to 43.8)	44.2 (34.6 to 53.8)	12.0 (5.6 to 18.4)	16.4 (9.3 to 23.4)	19.0 (12.0 to 26.1)
Ultimate PCI (Any time, All Patients)	–	–	85.6 (81.0 to 90.2)	69.8 (62.3 to 77.3)	93.2 (90.2 to 96.1)	54.1 (41.7 to 66.6)	91.9 (87.3 to 96.5)	77.9 (72.5 to 83.4)	49.2 (38.0 to 60.4)	30.4 (19.0 to 41.8)
Ultimate PCI (Any time,% of those having CA)	–	–	93.3 (89.8 to 96.7)	75.1 (68.4 to 81.7)	94.7 (92.1 to 97.4)	59.6 (49.2 to 70.0)	96.1 (93.6 to 98.6)	81.8 (78.1 to 85.5)	72.7 (63.4 to 81.9)	37.5 (26.7 to 48.2)
TLT & PCI (% patients having CA & PCI)	–	–	32.13 (25.6 to 38.6)	21.52 (12.3 to 30.7)	22.21 (16.5 to 27.9)	26.10 (20.3 to 31.9)	43.97 (34.5 to 53.5)	9.51 (3.3 to 15.7)	10.64 (4.5 to 16.8)	21.90 (12.6 to 31.2)

*For non-PCI hospitals in the ‘All Hospitals’ calculation, this is TLT at any time among patients.

CA, coronary angiography; PCI, percutaneous coronary intervention; STEMI, ST-elevation myocardial infarction; TLT, thrombolytic therapy.

**Table 4:** Angiogram and PCI use among patients with STEMI in PCI centres by sex and pre-hospital delay

		Pre-hospital delay (All STEMI)					Early phase STEMI				Evolved STEMI				Recent STEMI			
		<12	12–48 hours	>48 hours	Total	χ^2^, p-value	F	M	Total	χ^2^, p-value	F	M	Total	χ^2^, p-value	F	M	Total	χ^2^, p-value
Had angiogram?																		
No	n	31	3	5	39	4.25, 0.091*	8	23	21	0.30, 0.586	1	2	3	0.52, 0.443*	1	4	5	0.00, 1.000*
	%	6.9	2.4	9.1	6.2		8.1	6.5	6.9		4.6	1.9	2.4		9.1	9.1	9.1	
Yes	n	421	122	50	593		91	330	421		21	101	122		10	40	50	
	%	93.1	97.6	90.9	93.8		91.9	93.5	93.1		95.5	98.1	97.6		90.9	90.9	90.9	
Total	n	452	125	55	632		99	353	452		22	103	125		11	44	55	
	%	100	100	100	100		100	100	100		100	100	100		100	100	100	
PCI use (among patients who had angiogram)																		
No	n	31	8	7	46	3.05, 0.235*	9	22	31	1.07, 0.301	4	4	8	6.46, 0.029*	2	5	7	0.38, 0.616 *
	%	7.4	6.6	14	7.8		9.9	6.7	7.4		19.1	4.0	6.6		20.0	12.5	14.0	
Yes	n	389	114	43	546		82	307	389		17	97	114		8	35	43	
	%	92.6	93.4	86	92.2		90.1	93.3	92.6		81.0	86.0	93.4		80.0	87.5	86.0	
Total	n	420	122	50	592		91	329	420		21	101	122		10	40	50	
	%	100	100	100	100		100	100	100		100	100	100		100	100	100	

*Fisher’s exact test.

PCI, percutaneous coronary intervention; STEMI, ST-elevation myocardial infarction.

### Treatment of patients with early phase STEMI

#### Angiography and PCI use

The vast majority of patients admitted to PCI hospitals within 12 hours of symptom onset received angiography and subsequent PCI, with no apparent differences by sex (table 4). Significant differences exist across the level of health facilities, with lower rates angiography in City hospitals and, inevitably, PCI, than in Federal and Regional hospitals. This pattern was consistent in both men and women (table 5). Four hospitals (two Federal and two Regional) performed angiography on all patients with early phase STEMI.

**Table 5:** Treatment received by patients with early phase STEMI in PCI centres, by sex and hospital

			Federal hospitals				Regional hospitals				City hospitals			
			Female	Male	Total	χ^2^, p-value	Female	Male	Total	χ^2^, p-value	Female	Male	Total	χ^2^, p-value
Initial TLT	No	n	20	77	97	0.68, 0.411	46	103	149	13.51, <0.001*	6	28	34	0.52, 0.470*
		%	76.9	68.9	70.3		83.6	56.3	62.6		60.0	71.8	69.4	
	Yes	n	6	35	41		9	80	89		4	11	15	
		%	23.1	31.3	29.7		16.4	43.7	37.4		40.0	28.2	30.6	
	Total	n	26	112	138		55	183	238		10	39	49	
		%	100	100	100		100	100	100		100	100	100	
Angiogram	No	n	0	1	1	0.23, 1.000*	4	7	11	0.99, 0.253*	4	15	19	0.00, 1.000*
		%	0.00	0.9	0.7		6.9	3.8	4.5		26.7	27.3	27.1	
	Yes	n	26	112	138		54	178	232		11	40	51	
		%	100	99.1	99.3		93.1	96.2	95.5		73.3	72.7	72.9	
	Total	n	26	113	139		58	185	243		15	55	70	
		%	100	100	100		100	100	100		100	100	100	
Initial PCI (% of those that has angiogram)	No	n	8	37	45	0.06, p1.000*	7	75	82	14.30, <0.00*	5	13	18	0.95, 0.329
		%	30.8	33.3	32.9		13.7	42.6	36.1		50.0	33.3	36.7	
	Yes	n	18	74	92		44	101	145		5	26	31	
		%	69.2	66.7	67.2		86.3	57.4	63.9		50.0	66.7	63.3	
	Total	n	26	111	137		51	176	227		10	39	49	
		%	100	100	100		100	100	100		100	100	100	
Ultimate PCI (% of those that had angiogram)	No	n	3	5	8	1.93, 0.173*	4	12	16	0.03, 0.769*	2	5	7	0.20, 0.641*
		%	11.5	4.5	5.8		7.4	6.7	6.9		18.2	12.8	14.0	
	Yes	n	23	107	130		50	166	216		9	34	43	
		%	88.5	95.5	94.2		92.6	93.3	93.1		81.8	87.2	86.0	
	Total	n	26	112	138		54	178	232		11	39	50	
		%	100	100	100		100	100	100		100	100	100	

*Fisher’s exact test.

PCI, percutaneous coronary intervention; STEMI, ST-elevation myocardial infarction; TLT, thrombolytic therapy.

Only 31 of the 452 patients with early phase STEMI did not receive an angiogram (online supplementary WA, table 2); over a third of these patients was admitted to one Regional hospital, Belgorod, and were treated with primary TLT only. TLT use, both standalone and together with PCI, was particularly high among males in Regional hospitals, and significantly higher than women. Of the remaining patients that did not receive an angiogram, a high proportion attended one hospital, Rostov, which in official figures also has the lowest rate of PCI use among hospitals in our study (online supplementary WA, table 1). The few patients who had CA without subsequent PCI either had contraindications (online supplementary WA table 3) or received TLT, which can be appropriate in certain circumstances (such as stenosis of <50%). These patients were evenly distributed among hospitals. Thus, the low use of PCI in Rostov seems due to reduced access to angiography.

10.1136/openhrt-2019-001134.supp1 Supplementary data

Looking at the combined effects of patient and admission characteristics on CA and subsequent PCI using logistic regression (table 6), there was no significant association between sex and having an angiogram or, subsequently, undergoing PCI. Age was not associated with pathway to hospital. There was, however, a much lower probability of receiving an angiogram if admitted to a City hospital compared with a Federal hospital, but among those who did have one, the odds of going on to a PCI, while lower, were not significantly so. A formal test of interaction found no evidence that the association of sex with having an angiogram varied by hospital level. Importantly, there was no reduction in the odds of receiving PCI among those admitted outside working hours. For all variables, however, the confidence intervals were wide.

**Table 6:** ORs for having angiogram and ultimate PCI among patients with early stage STEMI in PCI hospitals

	ALL early phase STEMI patients	Early phase STEMI patients that had angiogram
	OR of having angiogram /no angiogram (*95% CI*)	OR of having ultimate PCI/*no PCI (*95% CI*)
Male *(ref: female)*	1.15 *(0.46 to 2.88)*	1.44 *(0.61 to 3.42)*
Age 60–75 *(ref: 35–59)*	0.62 *(0.27 to 1.43)*	0.73 *(0.33 to 1.59)*
Direct route *(ref: indirect)*	0.38 (0.08 to 1.71)	0.50 (0.17 to 1.48)
Regional Level Hospital *(ref: Federal)*	0.16 (0.02 to 1.24)	0.88 (0.36 to 2.14)
City Level Hospital *(ref: Federal)*	0.02***(0.00 to 0.19)	0.46 (0.15 to 1.37)
Comorbid *(ref: no)*	0.79 (0.35 to 1.78)	0.92 (0.42 to 2.02)
Admitted 00:00-05:59 *(ref: 06:00-17:59)*	1.03 (0.26 to 4.08)	2.65 (0.59 to 11.91)
Admitted 18:00-23:59 *(ref: 06:00-17:59)*	0.61 (0.26 to 1.43)	1.34 (0.57 to 3.18)
Constant	445.29*** (32.81 to 6043.91)	21.06*** (4.48 to 98.85)
N	452	420†

*p<0.05, **p<0.01, ***p<0.001.

†One further case excluded due to missing data.

PCI, percutaneous coronary intervention; STEMI, ST-elevation myocardial infarction.

#### Route to hospital and treatment en route

The first medical contact of nearly all patients with early phase STEMI was EMS (online supplementary WA table 4). 88.8% of patients transported by EMS was given aspirin prior to arrival at hospital, while a further 4.6% had already taken it. Almost all the remainder were given it after arrival at hospital. The corresponding figures for patients with evolved STEMI were 91.3% and 4.4%. Among patients at PCI hospitals, a third received TLT, with the majority receiving TLT prior to arrival (online supplementary WA table 4). Pre-hospital TLT use was significantly higher among patients travelling indirectly to the PCI hospital (via non-PCI facility), more than double the proportion of patients travelling directly by ambulance (online supplementary WA table 4). Almost half of patients transported by EMS for over 60 km received TLT in the ambulance, significantly more than the 18% of patients transported across shorter distances (online supplementary WA table 5).

### Treatment of evolved STEMI in PCI hospitals

20% of patients with STEMI at PCI hospitals presented with evolved STEMI, the vast majority underwent angiography (tables 2 and 4). There was no sex difference and while all those without comorbidity underwent this procedure, the corresponding figure was slightly, though significantly, lower for those with comorbidity (93%, n=45) (table 7). Among patients having subsequent PCI, however, a sex difference was found; PCI was received by nearly all men having CA but only 81% of women (table 4). After adjusting for age and comorbidity, this difference remained; the OR for receiving a PCI among men relative to women was 5.25 (95% CI: 1.14 to 24.13) (online supplementary WA table 6).

**Table 7:** Angiography by age, sex and comorbidity in patients with evolved and recent STEMI PCI centres

Received angiography?			Age				Sex				Comorbidity?			
			35–59 years	60–75 years	Total	χ^2^, p-value	Female	Male	Total	χ^2^, p-value	No	Yes	Total	χ^2^, p-value
Evolved STEMI	No	n	1	2	3	0.74, 0.574*	1	2	3	0.52, 0.443*	0	3	3	5.46, 0.045*
		%	1.4	3.8	2.4		4.6	1.9	2.4		0.0	6.7	2.4	
	Yes	n	71	51	122		21	101	122		80	42	122	
		%	98.6	96.2	97.6		95.5	98.1	97.6		100	93.3	97.6	
	Total	n	72	53	125		22	103	125		80	45	125	
		%	100	100	100		100	100	100		100	100	100	
Recent STEMI	No	n	1	4	5	0.12, 0.144*	1	4	5	0.00, 1.000*	4	1	5	0.01, 1.000*
		%	3.5	15.4	9.1		9.1	9.1	9.1		8.9	10	9.1	
	Yes	n	28	22	50		10	40	50		41	9	50	
		%	96.6	84.6	90.9		90.9	90.9	90.9		91.1	90	90.9	
	Total	N	29	26	55		11	44	55		45	10	55	
		%	100	100	100		100	100	100		100	100	100	

*Fisher’s exact test.

PCI, percutaneous coronary intervention; STEMI, ST-elevation myocardial infarction.

#### Route to hospital and TLT use

The majority of patients transferred from non-PCI hospitals arrived at the PCI hospital within 24 hours (67%), of those that did not are unable to assess if this is due to later arrival at the non-PCI hospital or prolonged door-to-door transfer from non-PCI hospital to PCI hospital. The majority of patients with evolved STEMI travelling by ambulance did not receive TLT, in line with ESC guidelines.

### Treatment of recent STEMI in PCI hospitals

Approximately 1 in 11 patients with STEMI presented very late at PCI hospitals (table 2). 90% of patients with recent STEMI had CA. There were no significant differences in the odds of having angiography by sex, age or comorbidity, although the values of n were small. No patients with recent STEMI with comorbidities went on to have a PCI; there were no differences in the odds of having PCIs by age and sex (online supplementary WA tables 7 and 8).

### Treatment at non-PCI hospitals

40% of patients with MI at non-PCI hospitals had STEMI, a significantly lower proportion than in PCI hospitals (70% patients with MI were STEMI) suggesting that, in general, early phase STEMI are being transferred on to PCI hospitals after admission, but clearly not all (table 1). Among the 54 patients with STEMI in non-PCI hospitals, data on pre-hospital delay were available for 49 (table 2). Just under half of these patients presented with an early phase STEMI yet were not transferred to PCI hospitals. In addition, eight patients had been transferred into the non-PCI hospital from another non-PCI hospital. We were unable to determine reasons why these patients were not transferred to a PCI hospital. Data on treatment for early phase STEMI at non-PCI hospitals were available for 22 of 23 patients, half of whom received TLT. Treatment varied across the hospitals with all, or almost all patients in three of the four hospitals receiving no TLT, but 10 of 12 in a fourth received TLT (online supplementary WA table 9).

## Discussion

The main, and the most important, finding is that, among those patients reaching PCI hospitals included in this study and surviving 24 hours, the vast majority were investigated and treated in line with current ESC guidelines. Previously, we have documented the dramatic expansion in health facilities equipped for PCI across Russia.^7^ Our findings suggest that across a range of these mainly new PCI hospitals the needs of patients with STEMI that reach them on time, are largely met.

Notwithstanding the need for caution given the purposive nature of the study design, within those meeting our inclusion criteria, the majority of patients with STEMI in Russia present in the early phase and those who reach PCI hospitals at this stage and have angiography have a very high probability of PCI. Indeed, while a number of studies in countries other than Russia have found that compared with men, women with CVD receive suboptimal treatment and have higher risks of adverse outcomes, our study finds, among patients with early phase STEMI who can be expected to benefit most, no significant difference in PCI use by sex after adjusting for comorbidity, age and hospital type. This is in line with more recent research suggesting that the well-known sex disparities could be explained by differences in case mix.^14 15^ Moreover, consistent with ESC guidelines, patients who did not receive PCI after angiography tended to have multivessel disease or other contraindications, while PCI use did not vary according to the admission route taken by the patient or time of admission, suggesting that hospitals really do offer a 24-hour service. We did, however, find a sex difference in PCI use among patients with evolved STEMI.

Adherence to ESC recommendations did vary; we identified differences in angiography and PCI use across different levels of hospital. Although angiography rates are high overall in patients with STEMI, they are significantly lower in City hospitals in our sample. Moreover, patients with STEMI in City hospitals who have an angiogram are less likely to have PCI (table 3). Additionally, we identified one hospital, Rostov, as an outlier in our sample: nearly half of all patients with early phase STEMI do not receive an angiogram there. While official figures indicate that a very low proportion of patients arrive there within 24 hours, the MAMIR data highlight that even among patients who do arrive early, angiogram use is low. Pre-hospital delay alone does not explain the low use of angiography, and thus subsequent PCI, in these patients with STEMI. Further investigation revealed that even though this hospital does provide PCI, it is not the main hospital for PCI in the region. Thus, while it seems most patients with AMI are taken to another hospital if time and distance permits, improvements in treatment for those patients left behind are possible.

A key finding from the study is that a number of hospitals in our study performed particularly well in specific areas of AMI management that were in line with ESC guidelines. Indeed, four hospitals offered angiography to all patients with early phase STEMI. These hospitals could potentially serve as a beacon to others, offering scope for shared learning. Additionally, AMI management of patients with early phase STEMI is particularly good, while there remains room for improvement for evolved and recent STEMI, in particular, with sex differences in PCI use existing among patients with evolved STEMI. Inter-hospital knowledge transfer will be particularly important as PCI health facilities continue to expand across the Russian Federation, in addition to strategies to improve pre-hospital delays.

The challenge now is to ensure that those facilities that are lagging behind are brought up to the standards observed in the best. Since 2010, the Federal Ministry of Health has adopted a range of measures designed to improve quality of care. These include both additional resources for healthcare and various targeted new initiatives such as revised clinical practice guidelines, drawing on international standards, pay for performance schemes, electronic medical records and various quality control programmes. These have been described in a recent review, which documented a high level of commitment to better quality care but also very limited evaluations, reflecting the lack of capacity for health services research in Russia.^16^ However, a further challenge is the persisting siloed and hierarchical management structure in many Russian hospitals, something that is deeply embedded in the system and will be difficult to overcome.^17^

This study also provides some evidence on pathways tohospital and TLT use for patients with STEMI in these hospitals. Just over a quarter of patients arrived at hospital 12 hours after symptom onset. Pre-hospital delay is a complicated issue, which involves both health seeking behaviour (patient delays) and health system factors (transportdelays). Patient delays (prolonged delays fromsymptom onset to seeking help) can reflect many factors, from symptom recognition to proximity and access to care. A proper understanding will require detailed research on health seeking behaviour using qualitative and quantitative approaches.

A key finding of this study is that there is a largeproportion of patients with STEMIwhohave long travel times to PCI hospitalsbut do not receive TLT. The STREAM trial examined the effectiveness of TLT inthose unable to receive immediate PCI. Subjects were randomised to either delayed (median 3 hours) PCI or rapid administration of TLT with rapid PCI where that failed or otherwise at 6-24 hours. Those receiving TLT achieved as good cardiac outcomes as those undergoing primary PCI, although they experienced a slightly high rate of bleeding.^18^ Thus, in Russia, when patients are unable to obtain immediate PCIs butcan be transferred to a hospital where it is available, the administration of TLT is a tractable measure that could improve prognosis for patients with AMI.

In a separate study, we have mapped road travel times from all Russian districts to the nearest facility providing PCI in 2015 (both in the same region and in neighbouring ones).^8^ This identified two strategies that could reduce travel times and thus, hopefully, delays. The first was the creation of 67 new PCI centres, in addition to the 260 that were then operating. This would increase the share of the population within 60 min travel time by almost nine percentage points, benefiting 5.7 million people. The second was to permit people living near regional borders to attend the closest facility, wherever it was. However, this would increase the number of people within 60 min travel time by only 340 000.

Ultimately, in Russia as elsewhere, there is likely to be scope for integrated planning of services, spanning the entire patient journey. This has proven successful with the acute management of stroke, as in London and, in varying forms, some other places, and a similar approach is being evaluated in seven tertiary hospitals in Germany.^19–22^ However, the organisational challenges are considerable as this model has been difficult to implement elsewhere.

The results we have presented should be interpreted with caution as the MAMIR Study has several important limitations. These, to some extent, reflect the challenge of conducting health services research outside major hospitals. Our sample is intentionally diverse, covering multiple levels of the health system to describe contemporary management of patients with AMI in Russia. The hospitals included cannot, however, be taken as representative of the overall situation in Russia, as we were constrained in selecting only those where it was possible to gain commitment by a local co-investigator. Within this constraint, we did sample purposively to cover the geography of Russia and facilities at all levels within the health system. However, as we have shown previously, there is considerable variation in the extent to which regions have adopted advanced management of AMI^7^ and, taking the PCI rate per 100 000 in 2013 as an indicator of progress, we also span the entire range of those providing a service at that time. Thus, we included two regions in the top quintile of activity, and 4, 2, 3 and 2 in each quintile of declining activity.

Despite strenuous efforts, there was a relatively high level of missing data, including lack of information on pre-hospital delays in 96 patients with STEMI and we were unable to obtain detailed data that would have allowed us to understand overall patient flows within the hospital system and, specifically, how many patients arrived at a non-PCI hospital and were transferred. Nonetheless, the data from this study provide a much improved evidence base from which to assess the current management of patients with AMI in the Russian Federation.

## Conclusions

Our data provide a valuable snapshot of the management of AMI in the Russian health system. Our findings offer some grounds for reassurance as, once patients reach hospitals offering PCI, they do seem to receive treatment consistent with ESC recommendations. However, there are also areas that could be improved. First, there is greater scope to ensure consistent, standardised care is received by patients across health facilities (both within PCI and non-PCI hospitals), and, in particular, increasing access to angiography and subsequent PCI. Additionally, there is scope to increase use of pre-hospital TLT in those who could benefit in many of the regions included, particularly in patients transferred indirectly or over greater distances. However, a key finding of the study is that there are multiple hospitals in Russia that are providing high-quality care to patients with AMI. These hospitals can serve as beacons of good practice as PCI facilities continue to expand across Russia.

### Ethics statement

This main study and the pilot study was approved by the ethics committees at the National Research Institute for Preventive Medicine, Moscow, Russia (approval number 01-04/15 dated 03.02.2015) and at the London School of Hygiene & Tropical Medicine, London, UK (approval number 9993 dated 1 June 2015). All study participants signed informed consent to participate in the study, to grant the access to medical history and other medical documentation and to be contacted at 6 and 12 months after hospitalisation.
