# Supplementary material for: Management of patients with acute ST-segment elevation myocardial infarction in Russian hospitals adheres to international guidelines
Source: Open Heart. 2020 Jan 23;7(1):e001134. doi: 10.1136/openhrt-2019-001134 (PMC6999683; doi:10.1136/openhrt-2019-001134)
Supplement: Supplementary data [file openhrt-2019-001134supp002.pdf]

STEMI Type:	Early Phase STEMI	Evolved STEMI	Recent STEMI
Definition:	ST elevation on ECG<br>FMC* <12 hours of symptom onset	ST elevation on ECG<br>FMC 12-48 hours of symptom onset	ST elevation on ECG<br>FMC >48 hours of symptom onset
Treatment:	-Primary PCI within 2 hours of diagnosis by experienced team<br>-If PCI within 2 hours not possile, TLT should be commenced within 10 minutes<br>-After successfull fibronolysis, routine PCI within 2-24 hours after TLT<br>-Rescue PCI inficated for failed fibrinolysis	-Primary PCI if indicated where sympoms suggestive of ischaemia<br>-For others, routine PCI should be considiered	-Routine PCI not indicated in asymptomatic patients presenting after 48 hours
