# Supplementary material for: Management of patients with acute ST-segment elevation myocardial infarction in Russian hospitals adheres to international guidelines
Source: Open Heart. 2020 Jan 23;7(1):e001134. doi: 10.1136/openhrt-2019-001134 (PMC6999683; doi:10.1136/openhrt-2019-001134)
Supplement: Supplementary data [file openhrt-2019-001134supp001.pdf]

Web appendix

Figure 1 ***Definition of STEMI phases and recommended intervention***

See "Web Appendix Figure 1.tif"

*FMC – First Medical Contact

Table 1 ***Clinic Level Indicators from Official Sources for Regions in the Sample****

Clinic	Administrative Level	# MI Patients 2015	AMI <24hrs	Mortality (% of MI patients)	Mortality first 24 hours (% MI patients)	% PCI first 24hrs	% TLT first 24 hrs	# Patients receiving PCI in first 24 hours
Arkhangelsk	City	558	67.6	10.9	4.6	61.3	8.2	342
Barnaul	Regional	362	68.4	8.1	5.7	82.0	4.5	297
Belgorod	Regional	501	75.8	2.4	0.4	87.8	12.2	440
Bryansk†	Cardio Clinic	254	45.3	14.6	7.9	0	4.3	0
Kazan	Inter-regional	430	81.4	4.7	4.7	81.4	1.6	350
Kemerovo	Cardio clinic	1,032	65.0	9.5	4.8	68.1	3.9	703
Perm	City	1,052	83.6	9.1	3.5	66.7	8.0	702
Rostov	Emergency City	1,280	28.4	10.2	6.1	20.5	8.5	262
Samara 1	Regional	2,220	92.5	8.4	4.8	43.3	10.3	961
Tver 1	Regional	516	50.0	4.8	2.3	78.5	25.0	405
Tver 2‡	City	319	66.1	9.1	6.0	0	24.5	0
Tuymen	Regional	1,100	88.7	12.4	4.4	64.1	12.0	705
Khanty-Mansiysk	Regional	184	91.8	6.0	0.5	62.50	2.7	115

Source: Extracted from reports of Regional Health Departments (Form N14 Data on Clinical Performance)

*Data not available for additional clinics – Samara 2, Samara 3 (both non-PCI clinics) and Saratov

†No PCI available in 2015

‡ Non-PCI capable clinic

Table W 2 ***Thrombolysis use and reasons for no reperfusion among STEMI patients arriving in PCI-capable clinics within 12 hours of symptom onset, not receiving an angiogram***

		Arkhangel -[illegible]	Belgorod	Samara 1	Perm	Rostov	Saratov	Tuymen	K-M
**Total No angiogram**		3	5	2	1	16	1	2	1
**Received TLT**		3	5	1	1	1	1	1	1
**Total STEMI patients arriving within 12 hrs**		33	77	69	37	37	3	37	23
**Reasons Given for No Reperfusion Treatment**	None given/missing					8			
	Ineligible ECG								
	Treatment postponed					1		1	
	Too Late*					3			
	Patient Refused			1		3			
	Contraindications							1	

*inconsistent with symptom onset and admission date time data

Table W 3 ***Reasons for no reperfusion treatment in early phase STEMI patients who received an angiogram***

**Reason No Reperfusion Treatment Given**	**N**
Missing	6
Ineligible ECG	1
Postponed	1
Isolated occlusion of the apical segment of LAD	1
No coronary artery damage	1
Ineligible stenoses	2
Small diameter of vessels	2
Multiple coronary lesions	2

Table W 4 ***TLT use and Type of FMC among Early Phase STEMI patients having Medical Contact Prior to Arrival at PCI Centre***

**First Medical Contact**			**DIRECT to DHF**	**INDIRECT to DHF**		**Total**	
			FMC - Ambulance	FMC - Ambulance	FMC – Non-PCI Health Facility		Chi2
**TLT USE**	Pre-Hospital TLT	n	58	15	39	112	56.70, p0.000 P0.000 (Fisher)
		%	16.96	71.43	46.43	25.06	
	TLT In DHF	n	33	1	4	38	
		%	9.65	4.76	4.76	8.50	
	No TLT	n	251	5	41	297	
		%	73.39	23.81	48.81	66.44	
	Total	n	342	21	84	447	
		%	100.00	100.00	100.00	100.00	

Note: Among patients travelling by another health facility to the DHF who are transported to PCI centre by ambulance all received pre-hospital TLT received it in the ambulance to the no PCI centre and not in the non-PCI health facility from which they were transferred. 5 patients self-presented –for these patients FMC was the DHF

.

Table W 5 ***Pre-Hospital TLT use in Early Phase STEMI Patients travelling to PCI centre by Ambulance, by Distance***

		**Distance from where ambulance left for hospital and PCI clinics (km)**						**Chi2**
		up to 60km		>60km		Total		
		n	%	n	%	n	%	
**Received TLT in Ambulance**	No	276	82.39	15	53.57	291	80.17	13.49
	Yes	59	17.61	13	46.43	72	19.83	P<0.0001
	Total	335	100	28	100	363	100	
**Males**	No Pre-Hosp TLT	204	80.95	15	57.69	219	78.78	7.62
	Pre-Hosp TLT	48	19.05	11	42.31	59	21.22	P=0.006
	Total	252	100	26	100	278	100	
**Females**	No Pre-Hosp TLT	72	86.75	0	0	72	84.71	11.34
	Pre-Hosp TLT	11	13.25	2	100	13	15.29	P=0.001
	Total	83	100	2	100	85	100	P=0.022 (Fisher)
**Age 35-59 years**	No Pre-Hosp TLT	129	79.63	11	64.71	140	78.21	2.01
	Pre-Hosp TLT	33	20.37	6	35.29	39	21.79	P=0.156,
	Total	162	100	17	100	179	100	P=0.212 (Fisher)
**Age 60-75 years**	No Pre-Hosp TLT	147	84.97	4	36.36	151	82.07	16.60
	Pre-Hosp TLT	26	15.03	7	63.64	33	17.93	P<0.0001
	Total	173	100	11	100	184	100	P0.001 (Fisher)
**No comorbidity**	No Pre-Hosp TLT	181	81.53	9	50	190	79.17	10.04
	Pre-Hosp TLT	41	18.47	9	50	50	20.83	P=0.002
	Total	222	100	18	100	240	100	P=0.004 (Fisher)
**Co-morbidity**	No Pre-Hosp TLT	95	84.07	6	60	101	82.11	3.62,
	Pre-Hosp TLT	18	15.93	4	40	22	17.89	P=0.057,
	Total	113	100	10	100	123	100	P= 0.078 (Fisher)

Table W 6 ***Odds Ratios for receiving PCI among Evolved STEMI patients receiving Angiogram at PCI Centres***

	OR for Having PCI over Not Having PCI *(95% Confidence Interval)*
Age 60 to 75 *(ref 35-59 years)*	1.01 *(0.21-4.83)*
Male *(ref: female)*	5.25* *(1.14-24.13)*
Comorbid *(ref: no)*	0.66 *(0.14-3.18)*
Constant	5.3* *(1.12-25.11)*
N	122

**p<0.05*

Table W 7 ***Treatment of Recent STEMI patients***

	Age				Sex				Co-morbid?				Total	
	**35-59 years**		**60-75**		**Female**		**Male**		**No**		**Yes**			
	n	%	n	%	n	%	n	%	n	%	n	%	n	%
**No PCI**	5	17.2	7	26.9	3	27.3	9	20.5	11	24.4	1	10	12	21.8
**Received PCI**	24	82.8	19	73.1	8	72.7	35	75.5	34	75.6	9	90	43	78.2
**TOTAL**	29	100	26	100	11	100	44	100	45	100	10	100	55	100
**Chi2**	0.75, p=0.385				0.24, p=0.624, p=0.689 (Fisher)				1.00, p=0.317, p=0.430 (Fisher)					

Table W 8 ***Odds Ratio of Having PCI among Recent STEMI Patients receiving Angiography***

	**OR - Having PCI over Not Having PCI** *(95% C.I.)*
Age 60 to 75 *(ref 35-59 years)*	1.23 *(0.22-6.71)*
Male *(ref: female)*	1.87 *(0.28-12.50)*
Constant	3.48 *(0.51-23.9)*
N	50

Table W 9 ***TLT use in early phase STEMI patients that were not transferred to PCI clinics by Clinic***

		BRYANSK	SAMARA 2	SAMARA 3	TVER 7	Total	Chi
Received TLT	None	4	4	1	2	11	12.13, 0.007, 0.002 (fisher)
		100	80	100	16.67	50	
	TLT	0	1	0	10	11	
		0	20	0	83.33	50	
	Total	4	5	1	12	22	
		100	100	100	100	100	
